# 

**Published:** 1871-01

**Authors:** 


### Books and Pamphlets Received.

First Medical and Surgical Report of the Boston City Hospital. Edited by J. N. Borland, Physician, and D. W. Cheever, Surgeon. Boston: Little, Brown & Co.

The American Journal of Obstetrics and Diseases of Women and Children. Edited by E. Noeggerath, M.D., and B. F. Dawson, M.D. New York: Townsend & Adams, Vols. II. & III.

On Diseases of the Spine and Nerves. By C. B. Radcliffe, J. N. Radcliffe, J. W. Begbie, F. E. Ainstie, and J. R. Reynolds. London, Edinburgh, etc. Philadelphia: H. C. Lea, 1871. 8vo., pp196.

Satan in Society. By a Physician. Chicago: J. S. Goodman & Co. 12mo. pp412.

Report on Barracks and Hospitals, with Descriptions of Military Posts. Circular No. 4, War Department, Surgeon General's Office. 4to., pp493.

Approved Plans of Specifications for Post Hospitals. Circular No. 3, Surgeon General's Office.

The Physicians Hand Book for 1871. By Wm. and A. D. Elmer, M. D. New York: Townsend & Adams.

Beneficial Results from the Use of Mechanical Appliances in Pott's Disease of the Spine, illustrated with Cases. By Jacob A. Wood, M.D., of New York.

Bloodletting as a Therapeutic Resource in Obstetric Medicine By Fordyce Barker, M. D., Professor of Midwifery and Diseases of Women in the Bellevue Hospital Medical College, etc.

Vaccination and its Protective Power, in the State of West Virginia. By John C Hupp, M. D., State Vaccine Agent.

Retention of Urine depending on Stricture. By Alexander W. Stein, M.D., Professor of Physiology and Histology in the N. Y. College of Dentistry, etc.

Proceedings of the Texas State Medical Association at the meeting held in the city of Houston, June 15th, 1870.

Fifth Annual Report of the St. Catherines General and Marine Hospital, for the year ending July 31st, 1870, Ontario.

Annual Report of the Comptroller of the State of New York, for January, 1871.

New York Observer Year Book and Manual for 1871. Sidney Morse, Jr., & Co., 37 Park Row, N. Y. City.

Public Ledger Almanac for 1871. Philadelphia: Geo. W. Childs.

The Nation; Atlantic Monthly; Scribner's Monthly; N. Y. Observer; Avon Journal; Newspaper Reporter; Littel's Living Age.

